# Macrophage polarization in diabetic wound healing

**DOI:** 10.1093/burnst/tkac051

**Published:** 2022-12-29

**Authors:** Xingqian Wu, Wenjie He, Xingrui Mu, Ye Liu, Junyu Deng, Yiqiu Liu, Xuqiang Nie

**Affiliations:** College of Pharmacy, Zunyi Medical University, Zunyi 563000, China; College of Pharmacy, Zunyi Medical University, Zunyi 563000, China; College of Pharmacy, Zunyi Medical University, Zunyi 563000, China; College of Pharmacy, Zunyi Medical University, Zunyi 563000, China; College of Pharmacy, Zunyi Medical University, Zunyi 563000, China; College of Pharmacy, Zunyi Medical University, Zunyi 563000, China; College of Pharmacy, Zunyi Medical University, Zunyi 563000, China; Joint International Research Laboratory of Ethnomedicine of Chinese Ministry of Education, Zunyi Medical University, Zunyi 563000, China; Key Lab of the Basic Pharmacology of the Ministry of Education, Zunyi Medical University, Zunyi 563000, China; Cancer and Ageing Research Program, School of Biomedical Sciences, Queensland University of Technology, 37 Kent Street, Woolloongabba, Brisbane 4102, Australia

**Keywords:** Diabetic wounds, Macrophage polarization, Signaling pathways, Wound healing, Therapeutics

## Abstract

Impaired wound healing is one of the severe complications of diabetes. Macrophages have been shown to play a vital role in wound healing. In different wound environments, macrophages are classified into two phenotypes: classically activated macrophages and alternatively activated macrophages. Dysregulation of macrophage phenotypes leads to severely impaired wound healing in diabetes. Particularly, uncontrolled inflammation and abnormal macrophage phenotype are important reasons hindering the closure of diabetic wounds. This article reviews the functions of macrophages at various stages of wound healing, the relationship between macrophage phenotypic dysregulation and diabetic wound healing and the mechanism of macrophage polarization in diabetic wound healing. New therapeutic drugs targeting phagocyte polarization to promote the healing of diabetic wounds might provide a new strategy for treating chronic diabetic wound healing.

Highlights

- The different polarized macrophages play different roles in immune responses, tissue homeostasis and diabetic wound healing progression.
- The potential role of signal transduction pathways involved in macrophage regulation in diabetic wound healing and the role of gene and stem cell therapy in diabetic wound healing are summarized.
- Targeted regulation of macrophage polarization may be a new strategy for the treatment of diabetic chronic wound healing.

## Background

Diabetes-based metabolic syndrome has gradually become a significant chronic disease affecting human health. Impaired wound healing is the main complication of diabetes [1]. Chronic diabetic wounds can persist for months to years and often recur, leading to reduced quality of life and loss of skin and mucosal function [2]. The pathogenesis of diabetic wounds is complex and involves many different pathways. It is traditionally associated with the local hyperglycemic environment, advanced glycation end-product (AGE) accumulation, oxidative stress injury and chronic inflammation. However, increasing evidence suggests that macrophages play a pivotal role in diabetic wound healing [3]. Macrophage polarization is closely related to inflammation and wound healing. The inflammatory response in which macrophages participate is critical in the occurrence, progression and repair of diabetic wounds [4]. The polarization of macrophages’ two subtypes, M1 and M2, has different effects on the aggravation and regression of wound inflammation [5]. In normal wound healing, macrophages convert from M1 to M2 type. On the contrary, in diabetic wounds, the transformation of M1 to M2 macrophages is impaired, which is an abnormality related to the decrease of collagen in wound closure, angiogenesis and deposition of diabetic wounds [6]. This article aims to provide a new reference by reviewing the mechanism of macrophage involvement in wound healing and the regulation of phenotypic polarization in diabetic wound healing.

## Review

### Macrophage polarization

Macrophages are widely used in the body to maintain homeostasis and resist invasive pathogens [7]. They have long been recognized as potent immune effector cells with important roles in tissue homeostasis and injury [8]. Macrophages are powerful immune effector cells that play an essential role in tissue homeostasis and damage. Functional plasticity and diversity are among the distinguishing features of monocyte-macrophages [9].

The function of macrophages changes dramatically with changes in the surrounding environment. Therefore, activated macrophages can be broadly divided into two categories based on their function: classically activated macrophages (referred to as caMφ or simply M1 type), representing a highly pro-inflammatory state of activation, and alternatively activated macrophages (referred to as aaMφ or M2 for short), representing opposing anti-inflammatory and pro-reparative activation states. The activation of M1 type cells is mainly by interferon-γ secreted by CD4^+^ helper T cells (Th1), lipopolysaccharide (LPS) of Gram-negative bacteria, granule macrophage colonization factor, and mediated by tumor necrosis factor (TNF). They are manifested as enhanced self-antigen presentation ability, complement-mediated phagocytosis and several pro-inflammatory factors such as TNF-α), interleukin-1β (IL-1β, IL-6, IL-12, IL-23 [10], nitric oxide (NO) release and chemokine ligand 9 (CXCL-9) and CXCL-10 production. The release of these antimicrobial and antitumor inflammatory factors can mediate reactive oxygen species (ROS)-induced tissue damage and disrupt tissue regeneration and wound healing.

M2-type macrophages have distinct roles from M1-type macrophages. M2-type macrophages can suppress the inflammatory response and promote angiogenesis and tissue repair in inflammatory diseases. They are classified into three types based on the specific functional markers released after being activated: M2a, M2b and M2c. M2a, activated by IL-4 or IL-13, secretes many anti-inflammatory cytokines such as IL-10 and expresses the mannose receptor and the macrophage galactose type C lectin, promoting Th2 immunity. Some Toll-like receptor (TLR) ligands induce M2b, which secretes a massive concentration of IL-10 and minuscule levels of IL-12, inhibiting acute inflammation caused by bacterial endotoxin while promoting Th2 differentiation and humoral immunity. Open-loop steroids, IL-10, glucocorticoids M2c, IL-10 hypersecretion and transforming growth factor (TGF) hypersecretion are regulated and suppressed by hormones [11].

**Figure 1:**
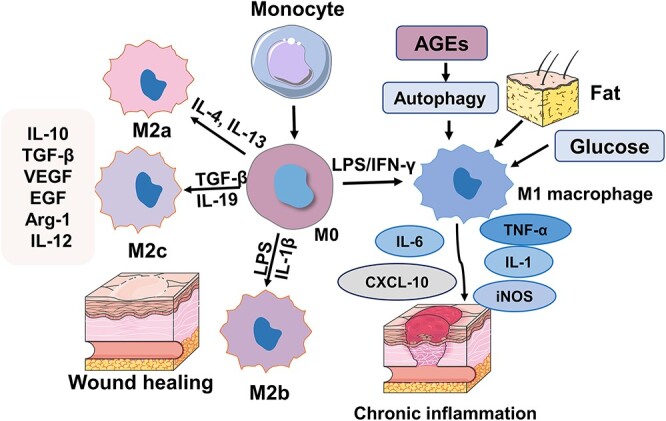
Macrophage polarization and wound healing. Macrophages are stimulated by the external environment and activate M1 or M2 macrophages. M1 macrophages secrete inflammatory factors, such as interleukin IL-1β, IL-12 and IL-6, which promote inflammation occurrence and development and delay tissue repair. M2 macrophages inhibit the occurrence and development of inflammation and promote tissue repair by synthesizing and secreting anti-inflammatory cytokines IL-10, arginase 1 (Arg-1), etc. *IFN-γ* interferon gamma, *TGF-β* transforming growth factor-beta, *IL* interleukin, *AGEs* advanced glycation end-products, *LPS* lipopolysaccharide, *VEGF* vascular endothelial growth factor, *iNOS* inducible nitric oxide synthase, *EGF* epidermal growth factor, *Arg-1* arginase-1

The molecular mechanism of M1/M2 macrophage polarization is unclear and the main related pathways are known as non-receptor tyrosine-protein kinase/signal transducer and activator of transcription (JAK/STAT), interferon regulator (IRF), Notch and phosphatidylinositol-3-kinase (PI3K/AKT) [12]. MicroRNAs (miRNAs) and long noncoding RNAs (lncRNAs) also play a critical role in macrophage polarization [13]. In addition, a hyperglycemic environment is one of the pathways that leads to an increase in pro-inflammatory cytokines, which in turn increases the polarization of M1 macrophages. For example, the same microenvironment with elevated glucose stimulates macrophages to secrete pro-inflammatory cytokines, such as IL-1, IL-6 and TNF-α, promoting a vicious cycle that maintains M1 macrophage polarization and chronic inflammation. In addition, obesity alone can lead to M1 macrophage polarization. This effect is amplified in the context of elevated plasma free fatty acids in obese patients, leading to increased inflammatory expression through the nuclear factor kappa-B (NF-κB) pathway [14]. A recent study reported that O-linked N-acetylglucosamine (O-GLC) acylation of c-Myc also plays an important role in megakaryocyte differentiation [15]. Autophagy regulates macrophage polarization and mammalian target of rapamycin (mTOR) pathway activation induces macrophage polarization. Rapamycin, a well-known inducer of autophagy that acts by inhibiting the mTOR pathway, has been demonstrated to stimulate the M1 phenotype of macrophages [16]. In addition, studies have also reported that AGEs promote the polarization of macrophages toward the M1 phenotype through autophagy activation, resulting in refractory wounds (Figure 1) [17].

### Macrophage polarization in diabetic wounds

Macrophages are involved in almost all stages of wound healing. After skin injury, monocytes aggregate and differentiate into macrophages on the wound surface. Macrophages infiltrating the wound site are activated to the M1 phenotype, marked by specific proteins such as CD86, inducible nitric oxide synthase (iNOS) and TNF-α, and mainly exhibit pro-inflammatory properties [18]. They can devour all the cell fragments of microorganisms, stroma and platelets, secrete pro-inflammatory mediators and chemokines, and recruit more circulating monocytes for phagocytosis, angiogenesis and re-epithelization [19,20].

Anti-inflammatory M2 macrophages exert anti-inflammatory effects and promote angiogenesis and collagen deposition to prevent delayed healing [21,22]. Diabetic wounds are M1-excessive in early stages but show M2-deficiency in later proliferative stages, suggesting that alterations in macrophage activation may contribute to impaired healing of diabetic wounds. Strategies to reverse this abnormal activation could be used to enhance the wound-healing process [23]. Therefore, promoting macrophage migration, phagocytosis and M2 polarization is of great significance for regulating the immune microenvironment of wounds and promoting wound healing. For example, treating diabetic mice with docosahexaenoic acid significantly improved ulcer healing by stimulating macrophage polarization toward the M2 phenotype [24]. Future research can be conducted to study the distribution of M1/M2 macrophages and the changes of cytokines in wound healing and scar formation [25]. Polarization of macrophages to an anti-inflammatory type significantly improved delayed wound healing. Therefore, various approaches for modifying the phenotype of macrophages can be used as therapies for treating chronic wounds.

The development of diabetic foot ulcers (DFU) is often due to abnormalities in the lower extremity neurovascular system, local microcirculation and oxygen metabolism. The specific mechanism of the refractory DFU is still unclear. In recent investigations, the prolonged inflammatory response and deregulation of macrophage phenotype have been linked to delayed healing of diabetic ulcers. Diabetic patients have high glucose levels, altered local microenvironment of ulcer and local ischemia and hypoxia. Macrophages are the central innate immune cells and are multifunctional cells that can regulate different processes in multiple stages of wound healing. In diabetic ulcers the abnormal cell phenotype of macrophages and the number and proportion of M1-type macrophages is increased. In a study of chronic skin ulcer wounds in diabetic mice, the high content of inflammatory factors in the wound hindered the transformation of M1 macrophages to M2 macrophages, resulting in excessive wound inflammation and delayed wound healing [26].

### Signaling pathways regulating macrophage polarization in diabetic wound healing

#### Notch signaling pathway

Notch proteins are found on the cell surface and have a long evolutionary history in vertebrates and invertebrates [27]. A transmembrane receptor group comprises the Notch external domains, the Notch internal region and the membrane domain composition. Notch receptor precursors are hydroxylated by O-fucosyltransferase in the Golgi apparatus, subsequently digested by gene duplication (acting at the S1 site) in the transporters Golgi, forming non-covalent heterodimers which are then transported to the cell membrane to become mature transmembrane protein receptors [28]. The Notch-recombinant signal-binding protein Jk (Notch-RBP-J) signaling pathway is indispensable in macrophage development and activation and regulates macrophage polarization [29]. Notch signaling is a critical regulator of macrophages’ biological function. The Notch signaling pathway includes four Notch receptors (Notch1–4) and five ligands (Ddltal1, 3, 4, Jagged1–2). Although Notch4 expression is restricted to mature macrophages, pancreas and epithelial cells, macrophages express Notch ligands and receptors (Notch1, 2, and 4), implying that Notch signaling is implicated in cytotoxic activity and regulates multiple biological properties [30]. LPS can selectively upregulate Notch1 expression by engaging macrophage monocyte pathways other than the previous 88 [myeloid differentiation factor 88 (MyD88)]-dependent or autonomous. Notch signaling enhances the production of inflammatory molecules IL-6 and iNOS, decreases the secretion of IL-10 and alienates macrophages toward M1 when activated. Notch activation suppresses signal-regulatory protein alpha (SIRPα) expression through hairy and enhancer of split (HES) family co-repressors and miR-148a-3p-mediated Notch signaling to promote the production of inflammatory cytokines. ROS may also promote the polarization of macrophages toward the M1 phenotype. SIRPα can promote macrophage M2 polarization, and the major pathway of SIRPα is mediated by SHP-1 activation, further affecting NF-κB and AKT signaling and modulating macrophage immunosuppression [31].

Recent research reveals that blocking Notch via RBP-J disruption biases tumor-associated macrophage (TAMs) toward M2 polarization, promoting the progression of inflammatory diseases [32]. Notch signaling mediated by RBP-J and TLR signaling play a synergistic role in the regulation of macrophage function. Notch signaling on macrophages is regulated by the expression of its downstream genes *Hes1* and *Hey1* make adjustments [33]. A study has proved that J-dependent classical Notch pathway activator can induce the high expression of interferon regulatory factor 8 (IRF8) through the map kinase interaction kinase-eukaryotic translation initiation factor-interleukin-1 receptor-related kinase 2 (MNK1-eIF4E-IRAK2) pathway, thus inducing the expression of polarization-related molecular markers and promoting the inflammatory reaction in macrophages [29].

Mice with myeloid-specific Notch signaling deficiency (DNMAML^floxed^Lyz2^Cre+^) exhibited delayed early healing (days 1–3) and reduced inflammatory gene expression by wound macrophages. Notch receptor expression dramatically increased in wound macrophages in a rat with physiological type 2 diabetes mellitus (T2DM) since day 6 after the initial inflammatory stage of wound healing, which correlates with the increased production of inflammatory cytokines. Increased Notch1 and Notch2 were also observed in human monocytes from T2DM patients. Furthermore, in prediabetic mice with genetic defects in Notch signaling (DNMAML^floxed^Lyz2^Cre+^ on a high-fat diet), improved wound healing was observed at late time points (days 6–7). These findings suggest that Notch is important during the early stages of wound healing and directs the production of macrophage-dependent inflammatory mediators. In addition, in bone marrow-specific Notch signaling-deficient mice (DNMAML^floxed^Lyz2^Cre+^) with suppressed Notch signaling, early wound macrophage numbers were reduced and wound healing was delayed [34].

Experiments revealed that inhibiting Notch signaling lowered macrophage production of inflammatory cytokines, indicating that Notch is involved in wound macrophage phenotype and inflammation *in vivo*. Notch signaling is disrupted in diabetic wound macrophages. Another study pointed out that Notch signaling may be involved in forming M1 macrophages through some epigenetic mechanisms. For example, the expression of Notch-1 in macrophages is regulated by LPS, and LPS activates the expression of Notch downstream genes *Hesl* and *Deltex* through MyD88-dependent or independent pathways [35]. LPS, TNF-α and other M1-type inducing factors cause macrophages to be polarized to M2 rather than M1. When Notch signaling is activated, macrophages are polarized toward M1 regardless of whether M1 or M2 inducers are administered. The activation of Notch ligands DLL-1, DLL-4 and their target genes *Hesl* and *Deltex* can promote the activation of macrophages.

**Figure 2:**
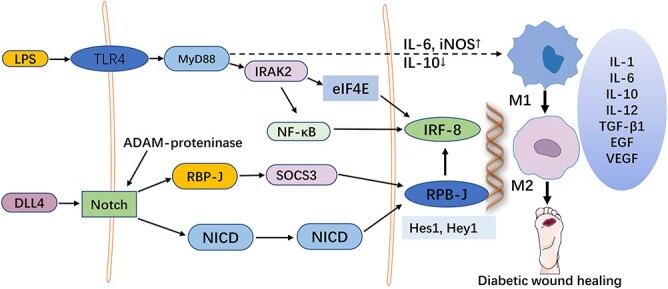
The role of Notch signaling in the regulation of macrophage polarization in diabetic wounds. When diabetic wounds occur, the Notch signaling pathway in macrophages is activated and its downstream target genes secrete stimulatory factors to polarize macrophages into M1 or M2 macrophages. The balance between M1/M2 macrophages is disrupted, resulting in prolonged inflammatory response at the wound site and delayed healing. *LPS* lipopolysaccharide, *TLR4* toll-like receptor 4, *MyD88* myeloid differentiation factor 88, *NICD* notch internal region, *RBP-J* recombinant signal-binding protein Jk, *DLL4* delta-like 4, *IRAK2* interleukin-1 receptor-associated kinase 2, *IRF-8* recombinant interferon regulatory factor 8, *eIF4E* eukaryotic initiation factor 4E, *NF-κB* nuclear factor kappa-B, *IL* inteleukin, *TGF-β* transforming growth factor-beta, *VEGF* vascular endothelial growth factor, *EGF* epidermal growth factor

Furthermore, the Notch signaling pathway can influence macrophage development indirectly by regulating the expression of macrophage surface marker CD11b [36]. Relevant studies have pointed out that inhibition of the Notch signal can reduce IL-1β and TNF-α secreted by M1 macrophages, thus reducing the level of inflammatory reaction. Therefore, inhibition of the Notch pathway will also become a direction of diabetic ulcer treatment [37] (Figure 2).

#### NF-κB signaling pathway

The NF-κB signaling pathway is a classical pathway that regulates macrophage polarization. The NF-κB signaling pathway involves a series of inflammatory and immune response processes. NF-κB plays a fundamental role in the inflammatory phase of wound healing by producing inflammatory mediators and vascular endothelial growth factor, an angiogenic cytokine. Various mediators mediate the inflammatory phase, mainly NF-κB and matrix metalloproteinase (MMPs) [38]. As an important transcriptional regulator, NF-κB is essential in synthesizing TNF-α [39]. Activation of NF-κB inhibitors promotes the phosphorylation of IκBα, followed by activation of NF-κB p65 and translocation into the nucleus to activate the expression of target genes [40]. Activating the Notch signaling pathway can activate the NF-κB signaling pathway. Under hyperglycemia, NF-κB activation is continuous, and the synthesis of AGEs is accelerated. This results in the inability of cells to remove AGEs on time, accumulation of AGEs in the body, damage to cells and tissues and an increase in local TNF-α levels, which impedes wound surface healing, suggesting that treatment of wound healing can be improved by activating the NF-κB pathway [41].

The binding of AGEs to the receptor for AGEs induces the production of intracellular ROS, which in turn leads to the activation of the nuclear transcription factor NF-κB, an inducer of pro-inflammatory gene expression. Enhanced levels of AGEs consistently trigger pro-inflammatory signaling by inducing macrophage M1 polarization, which disrupts the transition from inflammation to the next proliferative phase in diabetic wound healing [42]. pn282987 was found to be capable of preventing AGE-induced NF-κB activation [43]. This study revealed that pnu282987 reduced TNF-α production by inhibiting AGEs to reduce their stimulation of macrophages, and significantly inhibited AGE-induced macrophage NF-κB activation and receptor for advanced glycation end-product expression [43]. In mouse macrophage cell line RAW264.7 experiments, lucidone significantly increased the expression of NF-κB p65 and subsequently degraded its inhibitory protein IκBα. Lucidone activates NF-κB signaling in inflammation and proliferation in wound healing [44]. Overexpression of miR-146a in diabetes macrophages improves M2 macrophage polarization by inhibiting the TLR4/NF-κB axis, thereby improving wound healing in diabetic ulcers. Additionally, the function of miR-146a in the diabetic healing process *in vivo* was studied in a diabetic ulcer animal model, and the findings revealed that miR-146a increased diabetic ulcer wound healing via blocking the TLR4/NF-κB axis [45].

The angiogenesis inhibitor kallikrein-binding protein (KBP) inhibits diabetic wound healing by blocking angiogenesis. Using blood KBP concentrations and monocyte counts, researchers employed enzyme linked immunosorbent assay (ELISA) and flow cytometry to assess monocyte-macrophages in diabetes patients. In people with diabetes with DFU, the number of monocyte-macrophages is elevated and higher than in diabetic individuals without DFU [46]. KBP delayed the wound healing of normal mice, and blocking KBP could accelerate the wound healing of diabetic mice. KBP increased the number of pro-inflammatory M1 macrophages in diabetic wounds and decreased the infiltration of M2 macrophages, especially in the late stage of wound healing, which led to the continuous inflammation of diabetic wounds. It also activates the Notch 1/RBP-/Hes1 signaling pathway, which then upregulates iNOS. In addition, KBP promotes the phosphorylation and activation of NF-κB p65 and the translocation of p65 to the nucleus.

Furthermore, KBP suppressed the expression of cylindromatosis tumor suppressor protein, a deubiquitinating enzyme and a negative regulator of NF-κB signaling. *Hes1* downregulates cylindromatosis tumor suppressor protein and activates the IkB kinase (IKK)/IκBα/NF-κB signaling pathway. The effects of KBP on NF-κB signaling pathway activation were inhibited by the NF-κB signaling inhibitor JSH23 and the Notch signaling inhibitor (DAPT), whereas *Hes1* overexpression activated the NF-κB signaling pathway. KBP modulates the number and polarization of monocyte-macrophages by activating Notch signaling and cross-activating the NF-κB signaling pathway, thereby delaying wound healing [47].

#### PI3K/AKT signaling pathway

The PI3K/AKT signaling pathway regulates macrophage survival, proliferation and migration, and is associated with macrophage polarization [48]. Loss of the PI3K/AKT/mTOR signaling pathway initiates the development of several diseases, including cancer and its progression, obesity, cardiovascular disease and diabetes [49]. The PI3K/AKT/mTOR pathway is critical for cell migration, and its diminished function hinders epithelial-mesenchymal transition (EMT), cell proliferation and wound healing [50]. AKT is a serine/threonine kinase that serves as a signaling hub for various cellular functions. The activation of PI3K-dependent AKT also influences the activity of several downstream pathways involved in cell proliferation, angiogenesis, senescence, apoptosis and cell survival [51]. PI3K consists of a series of lipoprotein kinases that are classified according to their ability to activate the inositol ring of hydroxyl (3′-OH) in inositol phospholipids [52]. The PI3K/AKT pathway is activated by TLR4 and other disease receptors, cytokines, inflammatory cytokines and Fc receptors [53], which regulate downstream signals that control cytokine production.

**Figure 3:**
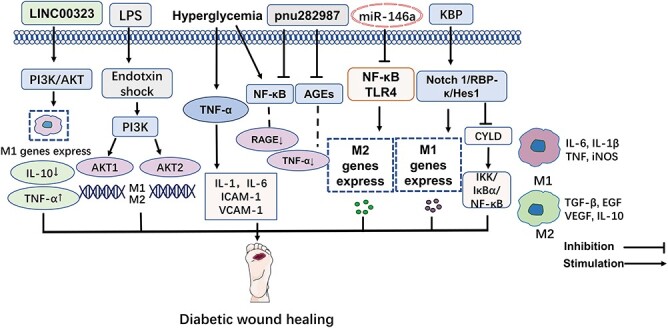
NF-κB and PI3K/AKT signaling pathways affect macrophage polarization in diabetic wounds. The PI3K/AKT/mTOR pathway is crucial for cell migration, and activation of the PI3K/Akt pathway can regulate macrophage polarization, participate in inflammatory responses and affect cell proliferation and wound healing in diabetic wounds. Under hyperglycemia, the activation of NF-κB persisted, the stimulation of the NF-κB signaling pathway promoted the polarization of M1 macrophages and the inhibition of NF-κB signaling pathway promoted the polarization of M2 macrophages. When drugs that improve the continuous activation of NF-κB act on chronic wounds, they can regulate the release of inflammatory factors during wound healing and promote the polarization of M2 macrophages by inhibiting the NF-κB axis, thereby promoting wound healing in diabetic ulcers. *TNF-α* tumor necrosis factor- α, *AGEs* advanced glycation end-products, *RAGE* receptor for advanced glycation end-product, *KBP* kallikrein-binding protein, *CYLD* cylindromatosis tumor suppressor protein, *TLR4* toll-like receptor 4, *ICAM-1* intercellular cell adhesion molecule-1, *VCAM-1* vascular cell adhesion molecule-1, *NF-κB* nuclear factor kappa-B, *IL* inteleukin, *TGF-β* transforming growth factor-beta, *VEGF* vascular endothelial growth factor, *EGF* epidermal growth factor, *iNOS* inducible nitric oxide synthase

Activation of the PI3K/AKT pathway exerts an anti-inflammatory effect in TLR-stimulated macrophages and is a negative regulator of TLR and NF-κB signaling in macrophages [54]. Activation or overexpression of PI3K or AKT kinases results in decreased LPS stimulation of macrophages, while nonspecific chemical inhibition of PI3K signaling in TLR-activated cells enhances NF-κB activation and iNOS expression to promote the response of M1-type macrophages [55]. Inhibiting AKT activity in macrophages promotes M1 polarization, whereas PI3K and AKT activation promote IL-4-induced M2 activation. Phosphatase and tensin homolog (PTEN) significantly enhances AKT signaling and induces the production of M2 macrophage markers [56]. The SH2-containing inositol 5′-phosphatase (SHIP, also called SHIP1) is a negative regulator of PI3K/AKT signaling, and SHIP-deficient macrophages favor M2 phenotype polarization and reduce inflammatory cytokine production [57]. It was found that PI3K, AKT, phosphorylated p-PI3K and p-AKT changed in different degrees in the skin damaged mouse model, and P-PI3K and p-AKT were expressed in inflammatory and proliferative phases. The expression of PI3K and AKT reached the peak in remodeling stage. This observation confirmed the important relationship between PI3K/AKT signal and wound healing [58]. However, AKT1 is considered a critical isoform sufficient to sustain cell growth compared to the AKT2 and AKT3 isoforms, both of which are required for normal cell growth and performance. An experiment on diabetic burn wounds showed that insulin-regulated macrophages switch from M1 to M2 phenotype and participate in anti-inflammatory effects through the PI3K/AKT signaling pathway, downregulating inflammatory responses and thereby improving chronic wound healing [59].

A key target (LINC00323) closely related to the PI3K/AKT signaling pathway was further explored at the level of cellular and molecular biology [60]. It was found that LINC00323 is also involved in the mechanism of M1 macrophage polarization. Furthermore, it was discovered in animal models that LINC00323 mediates M1 macrophage polarization via the PI3K/AKT signaling pathway and that inhibiting LINC00323 expression can alleviate the damage caused by diabetic nephropathy, which plays a crucial role in disease development. In addtion, lucidone accelerates wound healing by stimulating the PI3K/AKT, Wnt/β-catenin and NF-κB signaling cascades, through synergistic actions of keratinocyte/fibroblast/endothelial cell proliferation and migration (Figure 3) [61].

#### Other signaling pathways

In an STZ-induced diabetic mouse trauma model, melatonin-pretreated exosomes derived from mesenchymal stem cells (MT-Exo) effectively inhibited the pro-inflammatory factors IL-1β and TNF-α, while the relative expression of anti-inflammatory factor IL-10 was increased. *In vivo*, an enhanced M2 to M1 polarization ratio facilitated diabetic wound healing while reducing the inflammatory response, angiogenesis and collagen formation. By raising the ratio of M2 polarization to M1 polarization via stimulating the PTEN/AKT signaling pathway, MT-Exo can enhance diabetic wound healing by reducing the inflammatory response [62].

Activation of the Wnt/β-catenin signaling pathway plays a vital role in the proliferative phase of wound healing. At the molecular level, β-catenin is a subunit of the cadherin complex and is considered a component of the canonical Wnt signaling pathway [63]. Wnt plays a specific role in the regulation of beta-catenin function, and the Wnt/β-catenin signaling pathway is critical in wound healing by interacting with a series of molecules necessary for cell maintenance, motility, proliferation and re-epithelialization [64]. Lucidone activates the PI3K/AKT, Wnt/β-catenin and NF-κB signaling cascades to accelerate wound healing through the synergistic effects of keratinocyte/fibroblast/endothelial cell growth and migration and macrophage inflammation [44].

In a DFU mouse model study, WDR74 and M2 macrophages were reduced in the wound tissue of DFU mice. TGF-β/Smad pathway activation increased the expression of M2 macrophage markers (arginase-1 and YM1) and IL-4 and decreased the expression of the M1 macrophage marker [65]. TGF-β/Smad pathway activation also increased extracellular matrix (ECM) production and promoted wound closure in diabetic mice [66]. In another mouse experiment, IL-25 promoted M2 macrophage polarization and fibroblast activation dependent on PI3K/AKT/mTOR and TGF-β/Smad signaling, and intradermal injection of IL-25 improved diabetic mice’s lower wound healing rate [67].

The cyclic GMP-AMP synthase stimulator of interferon gene (cGAS-STING) signaling pathway has been identified as a prominent regulator of inflammation in sickness, cellular stress and tissue damage; its activation results in the overexpression of inflammatory genes. In addition, it activates signaling in the downstream direction [68]. The DNA in pathogens activates the cGAS-STING signaling pathway in macrophages, producing many cytokines in response to various stresses [69]. This signaling pathway can significantly regulate the polarization of macrophages. In an autocrine manner, it promotes the maturation and polarization of macrophages, enhancing their capacity for antigen presentation and cytokine secretion. The study discovered that palmitic acid (PA)-induced endothelial angiogenesis suppression is caused by disruption of the Hippo-Yes-associated protein (Hippo-YAP) system, an important signaling pathway governing tissue healing and regeneration, implying that PA disrupts the Hippo-YAP pathway and thus inhibits endothelial angiogenesis. PA induces the cytoplasmic release of mitochondrial DNA (mtDNA) and activates the cytoplasmic DNA sensor cGAS–STING–IRF3 pathway, thereby promoting mammalian Ste20-like kinases 1 (MST1) expression and inhibiting YAP and endothelial angiogenesis. This mechanism may be associated with impaired wound healing in diabetes. Interactions between signaling pathways may provide some new strategies for targeted therapy [70,71].

### Regulation of macrophage polarization promotes diabetic wound healing

Macrophages play a unique and irreplaceable role in diabetic wound healing, and the polarization of macrophages to anti-inflammatory types significantly improves delayed wound healing. Therapies targeting modulation of macrophage polarization have yielded beneficial results in diabetic wounds (Table 1).

**Table 1:** Potential drugs or therapies targeting modulation of macrophage polarization to promote diabetic wound healing

**Classification**	**Examples**	**Model**	**Effect on diabetic wound healing**	**Wound healing results**	**Reference**
Drugs	MT-Exo	Mouse (Rat)	Activate the PTEN/ATK pathway and decrease the number of M1 macrophages.	Wound healing is promoted	[62]
	Insulin	Rat	Induction of macrophage polarization from M1 to M2 phenotype.	Wound healing is promoted	[59]
	CSO	Mouse	IL-10, Arg-1↑	Wound healing is promoted	[72]
	pUBM	Mouse	Reduced inflammation via SHH pathway increases M2 polarization	Wound angiogenesis is promoted	[73]
	Quercetin, docosahexaenoic acid	Rat	Restoration of the M1/M2 balance of macrophages	Wound healing is promoted	[74,75]
	ON101	Mouse	Regulates the balance between M1 and M2 macrophages.	Wound healing is promoted	[76]
Biomaterials	Col I/SCS	Mouse	Facilitated the transition from M1 to M2.	Wound healing is promoted	[77]
	DESs	Human	Significantly increase the number of M2 macrophages	Wound healing is promoted	[78]
	HA-JK1	Cell	Facilitated the transition from M1 to M2.	Wound healing is promoted	[79]
	KSiNPs	Mouse	Induce differentiation to M2 phenotype.	Wound healing is promoted	[80]
	*Lactococcus lactis* thermo-sensitive hydrogel	Mouse	Promotes polarization of macrophages from M1 to M2.	Wound healing is promoted	[81]
Stem cells	ADSCs	Mouse	Induces macrophage polarization to M2 phenotype.	Wound healing is promoted	[82]
	MSC	Mouse	Regulates macrophage infiltration	Wound healing is promoted	[83]
	hMSCs	Rat	Induces macrophage polarization to M2 phenotype.	Wound healing is promoted	[84]
	BM-MSCs	Mouse	Promotes polarization of the anti-inflammatory M2 phenotype.	Wound healing is promoted	[85]
	ASCs	Mouse	Reduced the number of macrophages and the expression of pro-inflammatory cytokines.	Wound healing is promoted	[86]
miRNA and lncRNA regulation	miR-146a	Human	Inhibits the TLR4/NF-κB axis	Wound healing is promoted	[45]
	miR-145a-5p	Mouse	Blocks M1 polarization, promotes M2 activation	Wound healing is promoted	[87]
	miR-29ab1	Mouse	Modulate inflammatory response	Wound healing is promoted	[88]
	lncRNA GAS5	Mouse	Disable GAS5 function	Wound healing is promoted	[88]
	lncRNA XIST	Mouse	Enhanced fibroblast extracellular matrix production	Wound healing is promoted	[89]

*MT-Exo* melatonin-stimulated MSC-derived exosomes, *CSO* collagenase santyl ointment, *pUBM* porcine urinary bladder matrix, *Col I/SCS* sulfated chitosan (SCS)-doped collagen type I hydrogel, *DESs* dermal/epidermal substitutes, *HA-JK1* a new hybrid system formed by doping hydrogen sulfide and hyaluronic acid, *KSiNPs* KGM-modified SiO2 nanoparticles, *ADSCs* adipose tissue-derived stem cell, *MSCs* mesenchymal stem cell, *hMSCs* human mesenchymal stromal cell, *BM-MSCs* bone marrow mesenchymal stem cell, *ASCs* adipose tissue-derived mesenchymal stem cells

#### Drug and biomaterial therapy

Due to the characteristics of neuropathy and susceptibility to infection caused by diabetic ulcers, diabetic wounds often fall into a vicious cycle of abnormal inflammation and cannot be healed. The dysregulation of the macrophage phenotype aggravates the deterioration of the inflammatory response. Pharmacological approaches that target the modulation of macrophage polarization have been used in wound healing. Melatonin-pretreated exosomes derived from mesenchymal stem cells (MT-Exo) have been found to affect the polarization of macrophages in diabetic foot ulcers [62]. Insulin, a drug commonly used in the treatment of T2DM, has been found to have a significant effect on regulating macrophage polarization [59]; the application of insulin promotes the polarization of macrophages and the change from M1 to M2 phenotype, thereby accelerating wound healing in diabetic rats [90].

Collagenase santyl ointment (CSO) contains Clostridium collagenase, which not only provides enzymatic wound debridement, but is also found to up-regulate the expression of IL-10 arginase [72]. When applied to diabetic wounds, the modified collagen gel increased IL-10 expression and macrophage recruitment, and the number of M2 macrophages increased on the third and seventh days after treatment. Porcine urinary bladder matrix (pUBM) is considered a therapeutic agent because it not only reduces wound inflammation but also provides a scaffold for cell migration, adhesion and proliferation. In diabetic mice, subcutaneous injection of pUBM resulted in improved angiogenesis in chronic wounds by increasing polarization of M2 macrophages via the sonic hedgehog (SHH) pathway [73]. In addition, quercetin and docosahexaenoic acid can promote diabetic wound healing by regulating the balance of M1/M2 macrophages [74,75]. ON101 ointment can exert its therapeutic effect by regulating the balance between M1 macrophages and M2 macrophages. Further studies have shown that ON101 ointment can increase the polarization of macrophages to M2 type, and effectively accelerate wound healing in diabetes [76].

As a candidate for diabetic wound therapy, sulfated chitosan (SCS)-doped collagen type I (Col I/SCS) hydrogel is polarized to accelerate the resolution of excessive inflammation by reducing pro-inflammatory IL-6 production and increasing anti-inflammatory cytokines, including IL-4 and TGF-β1. Furthermore, the Col I/SCS hydrogel enhanced the differentiation of macrophages to fibroblasts, enhancing collagen and ECM formation in wound tissue [77]. Dermal/epidermal substitutes, which consist of a layer of bovine-derived type I collagen and a layer of silicone, when placed on freshly debrided DFUs, showed complete wound healing at 6 months in 60% of patients, compared to 20% in a control group. A significant increase in the number of M2 macrophages in the diabetic wound bed was noted on day 30 of treatment [78]. During the inflammatory phase of wound healing, the acidic microenvironment of a new dressing containing hyaluronic acid and H2S donor (HA-JK1) hydrogel accelerates the release of H_2_S, which leads to the polarization of macrophages from M1 to M2 type, which in turn terminates abnormal inflammatory responses over time and promotes wound regeneration [79]. konjac glucomannan-modified (KGM)-modified SiO_2_ nanoparticles efficiently activate macrophages to differentiate into M2-type phenotype by inducing mannose receptor clustering on the cell surface, using full-thickness ablation in diabetic or healthy mice models, thus significantly increasing closure rates [80].

Furthermore, the *Lactococcus lactis* thermosensitive hydrogel can be safely applied topically to the wounds of diabetic mice with full-thickness skin defects. It can promote the polarization of macrophages from M1 to M2, reshape the wound healing microenvironment and promote effective wound healing [81].

#### Stem cell therapy

As a new type of therapy, stem cell therapy mainly induces the differentiation of stem cells into insulin-secreting cells and treats diabetic skin wound ulcers by improving insufficient insulin secretion in diabetic patients. Adipose tissue is rich in stem cells, termed adipose tissue-derived stem cells (ADSCs), which have been reported to have great potential in wound repair and tissue regeneration. Phages polarize to the M2 phenotype to reduce the ability of macrophages to stimulate an inflammatory response, thereby reducing inflammatory responses in wounds [82]. In a skin injury model mimicking foot ulcers, mesenchymal stem cell (MSC) administration accelerated wound closure by modulating macrophage infiltration and restoring IL-1β and TNF-α mRNA to normal levels [83]. Human mesenchymal stromal cells (hMSCs) are a stem cell-based therapy. Repeated topical hMSC administration significantly accelerated wound healing in a diabetic rat model, which is beneficial for diabetic wound healing by improving angiogenesis and efficiently recruiting M2-like macrophages [84]. Bone marrow mesenchymal stem cells (BM-MSCs) can recruit macrophages to polarize toward an anti-inflammatory M2 phenotype with enhanced phagocytic capacity during wound healing in diabetic mice, thereby promoting wound healing [85]. Infusion of adipose tissue-derived mesenchymal stem cells (ASCs) in a diabetic mouse model reduced macrophage numbers and proinflammatory cytokines and increased expression of protective molecules that may help accelerate wound healing [86]. In addition, epithelial cells derived from placenta amnion (hAECs) have strong stem cell characteristics. Their exosomes, hAECs-Exos, can deliver miRNAs related to wound healing into human fibroblasts (HFBs) and human umbilical vein endothelial cells (HUVECs) and promote angiogenesis and fibroblast function by activating the PI3K-AKT–mTOR pathway, thus accelerating collagen deposition, vascular regeneration and diabetic wound healing in mice [91].

Stem cells have great potential in the treatment of diabetic wound healing. Many stem cell therapies that target and modulate the phenotypic properties of macrophages can restore the inflammatory homeostasis of the diabetic wound microenvironment and promote the healing of diabetic wounds. Stem cell technology is still mainly in the basic research stage and its clinical application is not yet mature. Understanding the detailed mechanism of stem cells in promoting wound healing will benefit many patients with chronic wounds that are difficult to heal in diabetic patients.

#### miRNAs and lncRNA regulation

miRNAs have a key regulatory function in macrophage development and polarization, according to several studies [92]. miRNAs are additional regulators of gene expression during macrophage polarization [87]. A group of studies showed that the expression of miRNA-146a (miR-146a) was elevated in ‘M2’ macrophages but attenuated in ‘M1’ macrophages. miR-146a exhibits protective effects on macrophages by inhibiting the activation and secretion of proinflammatory cytokines [45]. In the wounds of diabetic mice, the expression of miR-145a-5p was measured to determine its effect on macrophage polarization in mouse RAW 264.7 macrophages and wound healing in diabetic mice. We observed that miR-145a-5p blocks M1 macrophage polarization while promoting M2 phenotype activation, and using miR-145a-5p overexpression to correct macrophage polarization function accelerates diabetic chronic wound healing [88]. miR-29ab1 plays a crucial role in diabetes-related macrophage inflammation, and ectopic increases in miR-29a and miR-29b1 in diabetic skin wounds are associated with upregulation of M1 polarization and IL-1β, compared with healthy subjects, as well as with elevated TNF-α levels. miR-29ab1 is essential for diabetic wound healing by regulating the inflammatory response. miR-21 is essential for the inflammatory immune response, and in a mouse skin wound model, the binding of miR-21 to the target gene PDCD4 enhanced the activity of c-Jun-AP-1 and induced macrophages when large amounts of IL-10 anti-inflammatory cytokines are produced, thereby promoting wound healing.

Similarly, lncRNAs may also play a role in regulating macrophage polarization. lncRNAs are noncoding RNAs >200 nucleotides in length. In a study describing the role of lncRNAs in diabetic wounds, lncRNAs were found to play a fundamental role in increasing ROS production, and the relative levels of lncRNA GAS5 in wounds play a role in the wound healing response. Reduced levels of GAS5 in wounds appear to enhance healing by promoting the transition of M1 to M2 macrophages [89]. Another study found that lncXIST could enhance the polarization of M2 macrophages to promote burn wound healing by targeting the IL-33/miR-19b axis [93].

Targeted regulation of miRNA gene therapy shows good development prospects. However, due to the complexity of the mechanism of gene expression regulation in diabetes, the application of miRNA and lncRNA in the treatment of diabetic wound healing is still in the preliminary stage. Therefore, in future research, it is imperative to better understand the interaction between the body and the drug and provide more ideas for the further development of miRNA gene-drug therapy for diabetic wound healing.

## Conclusions

Macrophage polarization plays a critical role in diabetic wound healing. Uncontrolled inflammation and abnormal macrophage phenotype are important reasons hindering the closure of diabetic wounds. In order to achieve a better effect of promoting wound healing, novel materials and various hydrogel formulations can be used to modulate macrophage polarization in diabetic wounds, by stimulating macrophage polarization toward pro-reparative subtypes or by recruiting macrophage subpopulations before wound healing. Many new treatments can block inflammatory signals that impair wound healing and block the activation of M1 macrophages, thereby accelerating the healing of diabetic wounds. A growing body of research suggests that some potential drugs and therapies targeting the modulation of macrophage polarization signaling pathways may soon be new therapeutic avenues for treating diabetes and its complications including DFU. Inducing macrophages to switch from a pro-inflammatory M1 to an anti-inflammatory M2 phenotype may result in more effective relief in treating poor wound healing and scarring, making it a promising therapeutic strategy for preventing poor wound healing in diabetes.

## Abbreviations

AGEs: Advanced glycation end-products; DFU: Diabetic foot ulcers; ECM: Extracellular matrix; IL-1β: interleukin-1β; iNOS: Inducible nitric oxide synthase; IRF: Interferon regulatory factor 8; KBP: Kallikrein-binding protein; LPS: Lipopolysaccharide; miRNA: MicroRNAs; MSC: mesenchymal stem cell; MT-Exo: melatonin-pretreated exosomes derived from mesenchymal stem cells; MyD88: Myeloid differentiation factor 88; PI3K: Phosphatidylinositol-3-kinase; pUBM: Porcine urinary bladder matrix; ROS: Reactive oxygen species; SHIP: SH2-containing inositol 5′-phosphatase; SIRPα: signal-regulatory protein alpha; TGF-β1: transforming growth factor-β1; TLR: Toll-like receptor; TNF-α: Tumor necrosis factor α; T2DM; type 2 diabetes mellitus.
